# Evaluating the prophylaxis and long-term effectiveness of acupuncture for migraine without aura: study protocol for a randomized controlled trial

**DOI:** 10.1186/1745-6215-14-361

**Published:** 2013-10-30

**Authors:** Jiao Chen, Ling Zhao, Hui Zheng, Ying Li, Mingxiao Yang, Xiaorong Chang, Biao Gong, Yinlan Huang, Yanqin Liu, Fanrong Liang

**Affiliations:** 1 Chengdu University of Traditional Chinese Medicine, Chengdu, Sichuan, China; 2 Hunan University of Traditional Chinese Medicine, Hunan, Changsha, China; 3 Chongqing Medical University, Chongqing, China; 4 Ningxia Medical University, Ningxia, Gansu, China

**Keywords:** Study protocol, Pragmatic randomized controlled trial, Acupuncture, Migraine without aura

## Abstract

**Background:**

The instant-treatment effect of acupuncture for patients with migraines has been corroborated in numerous studies. However, most diseases are chronic and tend to recur, so the long-term effect of acupuncture can verify the existence of sustained efficacy or the placebo effect. Evaluating the efficacy of acupuncture in the prophylaxis of migraine without aura (MWoA) in China is also important because such studies are lacking.

**Methods:**

This trial is a multicenter, prospective, pragmatic randomized controlled clinical trial. We will randomly allocate 249 participants to three groups of 83. Patients in the individualized acupoint group will be treated with individualized acupuncture point prescriptions. The non-acupoint control group will undergo insertion of acupuncture needles at four bilateral non-points in locations not corresponding to acupuncture points. The waiting-list control group will not undergo treatment but instead will receive 20 acupuncture treatments for free after a waiting period of 24 weeks. Participants in the individualized acupoint group and non-acupoint control group will receive 20 sessions over four weeks and then all participants will receive 20 weeks of follow-up.

**Discussion:**

The results of our trial will help to supply evidence for the long-term acupuncture effect for MWoA in a long follow-up period, and special attention will be paid to comparison with the placebo effect.

**Trial registration:**

The trial was registered at ClinicalTrials.gov (NCT01687660) on 18 September 2012.

## Background

Migraine is a refractory disorder with high socioeconomic impact [1-5]. The prevalence of migraine among adults in the USA is approximately 28.4% [6] and 4.2 to 14.6% in China [7]. It has been listed as one of the most serious, chronic, and dysfunctional disorders with a prevalence equal to quadriplegia, mental disorders, and dementia according to the World Health Organization [8]. Several drugs can be used to reduce the frequency of migraine attacks: aspirin, acetaminophen and NSAIDs [3,9,10]. However, the success of treatment is usually modest and tolerability is often suboptimal [11].

Acupuncture is used widely in the prevention and treatment of migraine. Acupuncture is one of the main treatments of traditional Chinese medicine (TCM) and has been used for over 3,000 years. According to time course of its curative effect, acupuncture effect can be divided into 'instant’ and 'long-term’ effects [12]. In recent years, the instant effect of acupuncture for patients with migraine has been corroborated by numerous studies [13-18]. Most diseases are chronic and tend to recur, so the long-term effect of acupuncture can verify the existence of sustained efficacy or the placebo effect. The main factors affecting long-term effects have been hypothesized to be needling frequency per week and treatment duration [19].

Several randomized controlled trials (RCTs) in China and overseas have paid close attention to how long the effect is maintained after the last acupuncture session. Our previous studies on the effect of acupuncture on migraine showed that participants in the acupuncture group had fewer days with migraine compared with the control group during weeks 5 to 8, but that this difference was not significant. However, a significant reduction in the number of days with migraine during weeks 13 to 16 was noted [20]. In another clinical trial, patients were allocated randomly to receive ≤ 12 acupuncture treatments over three months or to a control intervention offering standard care: headache scores at three months and 12 months were lower in the acupuncture group than in controls. Those results suggested that acupuncture leads to persistent, clinically relevant benefits for patients with chronic headache (particularly migraine). That study with long-term follow-up concluded that the acupuncture effect for migraine prophylaxis can last nine months after treatment cessation [21].

We conjectured that there would be a long-term curative effect of acupuncture for migraine without aura (MWoA) over a longer follow-up period. We have designed a pragmatic trial to investigate the effectiveness and safety of acupuncture for the prophylaxis of patients suffering MWoA. In addition, special attention has been paid to a comparison with the placebo effect, accompanied by observation of the intensity and duration of the acupuncture effect.

## Methods

The design of this study is in accordance with the guidelines of the International Headache Society’s (IHS) Committee on Clinical Trials in Migraine [22].

### Design

**Figure 1:**
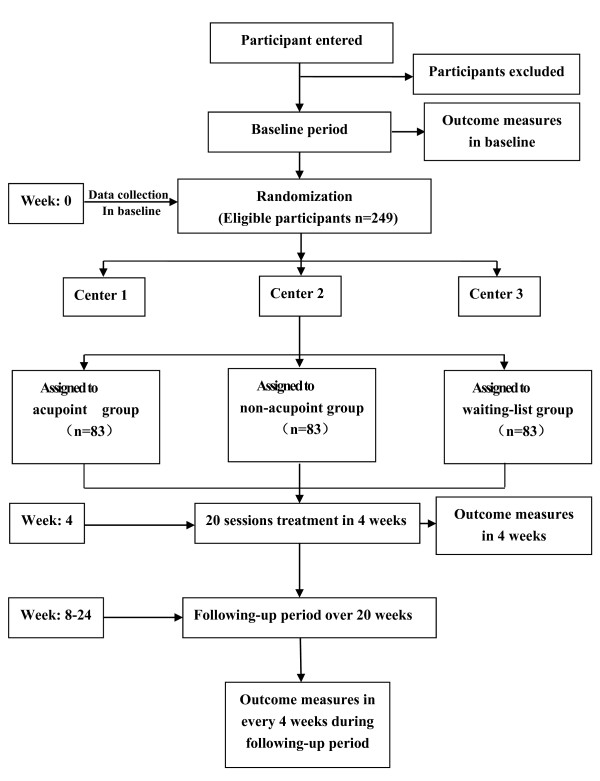
**Trial profile.** Participants with a diagnosis of migraine without aura will be recruited at three centers (enrollment areas of Chengdu, Hunan and Chongqing) taking part in the study. All participants should endure a baseline period of four weeks and inappropriate participants will be excluded. A total of 249 participants will be randomized to three groups: individualized acupoint, non-acupoint control, and waiting-list control. Each patient will receive four weeks of treatment and 20 weeks of follow-up. Outcome will be measured before randomization, the week of the last acupuncture session, and 8, 12, 16, 20 and 24 weeks after randomization, except that the acupuncture expectation value will be assessed before randomization, and MSQ, SAS and SDS will be assessed only at four weeks after treatment.

This randomized, controlled, multicenter pragmatic trial comprises three parallel groups. It aims to compare the effectiveness of the individualized acupoint group, non-acupoint control group (in locations not corresponding to acupuncture points), and the waiting-list control group (who receive delayed active acupuncture treatment 24 weeks later) (Figure 1).

In all groups, participants will not take any regular medications for migraines, but will be permitted to use necessary analgesics such as the triptans, ergotamines and analgesics during acute attacks of migraine. The type, dose and time of administration of the agent must be recorded in a headache diary.

The central randomization will be conducted using the Brightech-Magnasoft Clinical Information Management System (CIMS) (Address: 285 Davidson Avenue, Suite 504, Somerset, NJ 08873, Phone: 908-790-8888, Web Site: http://www.brightech-intl.com). Allocation to treatment groups uses a stratified block dynamic randomization method with permuted block, which is automatically under the control of a central computer system. To guarantee allocation concealment, randomization will be done by an independent researcher. The website and mobile message will be used to send randomization information (including the participants’ name in pinyin format, sex and date of birth) to the CIMS center. An independent assessor will interview the participants and carry out the screening. Random numbers and group assignment will be confirmed immediately through Email or short message service (SMS) to the independent assessor. This procedure guarantees that randomization concealment is adequate, and not influenced by the acupuncturists or participants. Participants allocated to individualized acupoint or non-acupoint groups will be blinded to their treatment allocation. However, blinding is clearly not possible in the waiting-list control group. We shall endeavor to ensure that participants begin the RCT with the same expectations of effectiveness by informing them that the treatments provided are effective. All participants will be assessed and the results will be analyzed by professionals blinded to the allocations of the different treatments. The duration of the study for every participant will be 28 weeks. Four of them will be before randomization (baseline), followed by four weeks of treatment, and finally 20 weeks of follow-up.

The Consolidated Standards of Reporting Trials (CONSORT) statement [23] (http://www.consort-statement.org/home/) has been used as a framework for developing the study methodology. The protocol of this study was approved by the Ethics Committee of the Teaching Hospital of Chengdu University of TCM (Chengdu, China) in June 2012 and is in accordance with the Declaration of Helsinki. The trial protocol has permission number 2012KL-007. The trial was registered in ClinicalTrials.gov with approval number NCT01687660. All participants have provided written informed consent to be included in the trial.

### Setting and participants

A total of 249 participants meeting the diagnostic criteria for MWoA according to the second edition of the IHS’ International Classification of Headache Disorders (ICHD-II, IHS 2004) will be recruited at three centers (Chengdu, Hunan and Chongqing) [24]. Participants will be: informed (verbally and through a print-out) of the possible risks associated with the study; instructed to complete the headache diary; told that they can withdraw from the trial at any time without specifying reasons; and told that they can voluntarily provide written informed consent before enrollment.

### Inclusion criteria

Eligible participants should match the diagnostic criteria for MWoA set by ICHD-II [24]. They must: (i) be male or female, aged between 18 and 65 years, with initial onset of migraines before the age of 50 years; (ii) have had two to eight migraine attacks, but less than fifteen days of attacks per month during the previous three months and during baseline measurement; (iii) suffering from acute migraine attack for ≥ one year; (iv) have completed the headache diary and given baseline values within it; (v) provided written informed consent by themselves or their next of kin.

### Exclusion criteria

Participants with any of the following conditions will be excluded: (i) headache caused by organic disorders such as subarachnoid hemorrhage, cerebral hemorrhage, cerebral embolism, cerebral thrombosis, vascular malformation, arteritis, hypertension or arteriosclerosis; (ii) neurological diseases, immunodeficiency, bleeding disorders and allergies. Those who have used prophylactic drugs in the previous month, pregnant women, women in lactation, and those planning to become pregnant within the next six months, and those involved in other RCTs, will also be excluded.

### Interventions

Treatment strategies were developed by consensus with experienced acupuncture practitioners and a neurologist. Based on TCM theory, a systematic review of the literature revealed that acupoints on the *Shaoyang* meridian were the ones chiefly selected for migraine [19]. There are three groups in this trial: individualized acupoint group, non-acupoint control group, and waiting-list control group. The location and manipulations of individualized acupoints and non-acupoints are shown in Table 1.

**Table 1:** Details of the acupoint and non-acupoint groups

**Group**	**Acupoint**	**Manipulation**
Individualized acupoint	(i) *Fengchi* (GB20)	(i) is punctured obliquely 0.8 to 1.2 cm toward to apex nasi
	(ii) *Shuaigu* (GB8)	(ii) is punctured horizontally 0.5 to 0.8 cm
	(iii) *Waiguan* (SJ5)	(iii) is punctured perpendicularly 0.5 to 1 cm
	(iv) *Yanglingquan* (GB34)	(iv) is punctured perpendicularly 1 to 1.5 cm
	(v) *Kunlun* (BL 60)	(v) is punctured perpendicularly 0.5 to 0.8 cm
	(vi) *Houxi* (SI3)	(vi) is punctured perpendicularly 0.5 to 0.8 cm
	(vii) *Hegu* (LI4)	(vii) is punctured perpendicularly 0.5 to 1 cm
	(viii) *Neiting* (ST44)	(viii) is punctured perpendicularly 0.5 to 1 cm
	(ix) *Taichong* (LR3)	(ix) is punctured perpendicularly 0.5 to 1 cm
	(x) *Qiuxu* (GB40)	(x) is punctured perpendicularly 0.5 to 0.8 cm
Non-acupoint control acupuncture	(i) At the medial arm on the anterior border of the insertion of the deltoid muscle at the junction of deltoid and biceps muscles	(i) is punctured perpendicularly 0.5 to 1 cm
	(ii) Half way between the tip of the elbow and axillae	(ii) is punctured perpendicularly 0.5 to 1 cm
	(iii) Ulnar side, half way between the epicondylus medialis of the humerus and ulnar side of the wrist	(iii) is punctured perpendicularly 0.5 to 1 cm
	(iv) Edge of the tibia 1 to 2 cm lateral to the *Zusanli* (ST36) horizontally	(iv) is punctured perpendicularly 0.5 to 1 cm

The individualized acupoint group *Fengchi* (GB20) and *Shuaigu* (GB8) are obligatory acupoints. GB20 will be punctured obliquely 0.8 to 1.2 cm toward apex nasi and GB8 horizontally 0.5 to 0.8 cm. Additional points can be chosen according to syndrome differentiation of meridians: (i) *Shaoyang* headache (headache only attacks the temporal side): *Waiguan* (SJ5) and *Yanglingquan* (GB34); SJ5 should be punctured perpendicularly 0.5 to 1 cm and GB34 perpendicularly 1 to 1.5 cm; (ii) *Taiyang* headache (headache involving the occiput): *Kunlun* (BL 60) and *Houxi* (SI3); BL60 will be punctured perpendicularly 0.5 to 0.8 cm and SI3 perpendicularly 0.5 to 0.8 cm; (iii) *Yangming* headache (headache involving the forehead): *Hegu* (LI4) and *Neiting* (ST44); LI4 is about to be punctured perpendicularly 0.5 to 1 cm and ST44 perpendicularly 0.5 to 1 cm; (iv) *Jueyin* headache (headache involving the vertex): *Taichong* (LR3) and *Qiuxu* (GB40), LR3 is intended to be punctured perpendicularly 0.5 to 1 cm and GB40 perpendicularly 0.5 to 0.8 cm.

In the non-acupoint control group, the four non-points are: (i) at the medial arm on the anterior border of the insertion of the deltoid muscle at the junction of deltoid and biceps muscles; (ii) half way between the tip of the elbow and the axillae; (iii) ulnar side, half way between the epicondylus medialis of the humerus and ulnar side of the wrist; (iv) The edge of the tibia 1 to 2 cm lateral to the *Zusanli* (ST36) horizontally. All of the four non-points ought to be punctured perpendicularly 0.5 to 1 cm.

#### *Individualized acupoint group*

In the individualized acupoint group, the acupoint prescriptions used will be personalized to each participant and at the discretion of the acupuncturist. Differentiating the location of meridians is an important part of TCM theory, so it was used to select acupoints on the basis of the evolution of the patient’s symptoms. *Fengchi* (GB20) and *Shuaigu* (GB8), having the highest frequency, were selected as obligatory acupoints. Additional points, which were used in a previous study [25], could be chosen according to syndrome differentiation of meridians: (i) *Shaoyang* headache (headache only attacks the temporal side): *Waiguan* (SJ5) and *Yanglingquan* (GB34); (ii) *Taiyang* headache (headache involves the occiput): *Kunlun* (BL 60) and *Houxi* (SI3); (iii) *Yangming* headache (headache involves the forehead): *Hegu* (LI4) and *Neiting* (ST44); (iv) *Jueyin* headache (headache involves the vertex): *Taichong* (LR3) and *Qiuxu* (GB40).

The acupuncturist responsible for the treatment will insert sterile, single-use filiform acupuncture needles (length, 25 to 40 mm; diameter, 0.25 mm) and auxiliary acupuncture needles (without manipulating the needles) of length 13 mm and diameter 0.18 mm after first disinfecting the skin with the participant lying down. The needles used are Hwato needles (Suzhou, China). The puncture will be made according to TCM standards to a depth of 0.3 to 1 cm depending on the points selected. Insertion will be followed by the stimulation methods of lifting and thrusting combined with twirling and rotating the needles to produce a sensation known as *Deqi*. Acupoints on the left and right side are employed alternatively, and punctured by filiform needles unilaterally. Auxiliary needles will be punctured 2 mm lateral to each acupoint to a depth of 2 mm without manual stimulation. This method has been used successfully in our previous study [20,26]. Han’s acupoint nerve stimulator (HANS; model LH 200A; TENS, Nanjing, China) will be connected after needle insertion.

Participants in this group will receive 20 sessions over a four-week period. Each session will be administered once a day for five continuous days followed by a two-day rest interval, and the participant will be connected to the stimulator for 30 minutes. All patients should complete ≥ ten treatment sessions. The stimulation frequency will be 2/100 Hz, and intensity varied from 0.1 to 1.0 mA until the participants feel comfortable. Needles will be retained *in situ* for 30 minutes and then the acupoint holes covered with clean cotton balls to avoid bleeding upon needle withdrawal.

#### *Non-acupoint control acupuncture*

Participants assigned randomly to this group will be given non-acupoint acupuncture, i.e., insertion of acupuncture needles at four bilateral non-points. The protocol of non-acupoints was developed in our latest acupuncture clinical trial [27]. The method will not differ from that used for the individualized acupoint group except for that an attempt will not be made to yield the *Deqi* sensation.

#### *Waiting-list control group*

No intervention will be used in the waiting-list group. The participants will be informed that they are scheduled to receive 20 acupuncture treatments for free after a waiting period of 24 weeks.

### Outcome measurement

The efficacy of acupuncture for migraine is assessed by the primary outcome measure: change in the frequency of migraine attacks during the 16th week after randomization.

The secondary outcome measures are: (i) frequency of migraine attacks; (ii) number of days with migraine; (iii) visual analog scale (VAS) score and grade of headache intensity (0 to 3); (iv) mean duration of migraine attack; (v) medication intake; (vi) number of participants with adverse events (AEs) and serious adverse events (SAEs); (vii) summary scales of the Migraine-Specific Quality-of-Life Questionnaire (MSQ) [20], Zung Self-Rating Anxiety Scale (SAS) [28] and Zung Self-rating Depression Scale (SDS) [28]; and (viii) acupuncture expectation value.

The outcome measures shown above will be measured before randomization, the week of the last acupuncture session, and 8, 12, 16, 20 and 24 weeks after randomization, except that the acupuncture expectation value is assessed before randomization, and MSQ, SAS and SDS are assessed only at four weeks after treatment.

Any AE and SAE, and how they are dealt with, will be recorded during the four treatment weeks and 20 follow-up weeks. AEs include bleeding, hematoma, fainting, severe pain, and local infection. Any SAE (including life-threatening SAEs) can lead to hospitalization or prolongation of existing hospitalization, and persistent or significant disability/incapacity. Therefore, intervention to prevent permanent impairment is required. If participants suffer AEs or SAEs, all details will be documented.

All physicians who enroll participants and assessors who collect data must attend training classes to ensure all practices at each hospital are identical. The training classes comprise theoretical and practical lessons. Physicians must pass the training test to understand the purpose and content of the trial, treatment strategies and quality control. Additionally, to maintain quality control, quality monitoring will be carried out by Brightech-Magnasoft CIMS, and specially trained physicians will check all trial processes. Detailed time points of outcome assessments are provided in Table 2.

**Table 2:** Timetable of treatment and outcome collection

**Period**	**Baseline**	**Treatment phase**	**Follow-up phase**				
Visit	1	2	3	4	5	6	7
Week	-4	1 to 4	8	12	16	20	24
Informed consent	×						
Demographic characteristics	×						
Medical history	×						
Laboratory tests, electrocardiography, pregnancy test	×						
Inclusion/exclusion criteria	×						
Vital signs	×	×	×	×	×	×	×
Change of medical history	×	×	×	×	×	×	×
Random allocation	×						
Acupuncture		⊗					
Headache diary	×	×	×	×	×	×	×
Visual analog scale	×	×	×	×	×	×	×
Headache intensity grade	×	×	×	×	×	×	×
Migraine-specific Quality-of-Life Questionnaire	×	×					
Zung Self-rating Anxiety Scale	×	×					
Zung Self-rating Depression Scale	×	×					
Acupuncture Expectancy Questionnaire	×						
Safety assessment							×

The detailed outcome assessment time points are provided. The outcome measures will be measured at baseline, the week of the last acupuncture session, and 8, 12, 16, 20 and 24 weeks after randomization, except that the acupuncture expectation value is assessed before randomization, and that MSQ, SAS and SDS are assessed only at four weeks after treatment.

× = all groups; ⊗ = treatment groups.

### Calculation of sample size and statistical analyses

Sample size will be estimated by NQuery Advisor v4.0 (Statistical Solutions, Boston, MA, USA). For this trial, it has been determined prospectively that α = 0.05 and 1-β = 0.90, and that the standard deviation will be 1.81 according to the three group subsets. According to a previous study [20], we anticipated that a mean frequency of migraine attacks in the non-acupoint group is 3.7 whereas, in the individualized acupoint group, it is 2.7. Hence, a minimum difference of clinical effect is 1.0. Thus, ≥ 70 participants are required for each group. To compensate for a prevalence of withdrawal of 15%, we plan to enroll 249 participants in the three groups, with 83 patients for each group.

For the final outcome analysis, we will make all pair-wise comparisons using a general linear model adjusted for baseline value, age, sex, clinical center, and disease course. The comparison between the individualized acupoint group and non-acupoint group is the primary interest in this study. In general, the summarization of difference is in accordance with CONSORT expectations, which will be addressed using effect size estimates and the associated confidence intervals.

All data in this trial will be assessed by Brightech-Magnasoft CIMS, with SPSS v13.0 (SPSS, Chicago, IL, USA) and SAS v9.1.3 (SAS, Cary, NC, USA). All analyses will be done on the intention-to-treat (ITT) population (that is, any participant randomized regardless of whether he/she receives any treatment). Missing data will be replaced according to the principle of multiple imputation. In addition, the per protocol (PP) population will be analyzed. The results of ITT and PP analyses will be compared to ascertain if the results are consistent. Moreover, analysis of variance (ANOVA) for repeated measures will be used for numerical variables. The Chi-square test will be used for categorical variables. *P* < 0.05 will be considered significant.

## Discussion

Pragmatic and explanatory randomized controlled trials play a significant part in the evaluation of healthcare interventions in China and overseas [29]. Explanatory trials are designed to ascertain whether a treatment has efficacy under ideal experimental conditions. Pragmatic trials are used to discover how effective a treatment is in routine everyday practice, with the aim of providing evidence that will help policymakers, practitioners or patients make choices between two interventions [30]. Pragmatic trials are used to ascertain if a treatment is beneficial under conditions close to those that operate in routine care, whereas effectiveness studies adopt a more pragmatic approach [31,32]. The greatest strength of pragmatic trials is that they can deliver evidence of effectiveness in the causal effects of a treatment [33].

The results of several trials suggest that an enhanced placebo effect impacts the acupuncture effect, which may explain patients’ positive beliefs and expectations of benefit of acupuncture and a benign acupuncturist-patient relationship [18,34-38]. We believe that this trial will demonstrate that the acupuncture effect is not due primarily to the physiological effects of acupoints. These influential factors have been standardized as strictly as possible and the selection of non-acupoints is different from those in previous studies. However, we cannot rule out the possibility that intervention using non-acupoint acupuncture may have some physiological effects. We designed a waiting-list group to ascertain if a psychological effect exists. There is no therapeutic intervention in the waiting-list group. Hence, we anticipate that the placebo effect from the acupuncturist-patient relationship and patients’ expectancy of benefit of acupuncture may be zero compared with the non-acupoint group. Furthermore, using parameters to attest to the specificity of acupoints, we will use non-acupoint stimulation in the control group. All participants will be followed-up.

The strength of our trial is strict central randomization, which will ensure adequate concealment of allocation and an equal chance of assignment in each group at all centers. Successful data collection from headache diaries and flexibility in treatment prescription will improve the compliance of practitioners. However, our trial will be limited by our inability to prevent patients knowing whether they are receiving treatment or waiting-list treatment. This factor may cause a high dropout rate in the waiting-list group because patients expect to receive acupuncture treatment when they join the trial. To prevent the expected high dropout rate, patients in the waiting-list group will receive 20 sessions of free acupuncture treatment after the entire course.

Briefly, the purpose of this trial is not to evaluate the efficacy of acupuncture as a painkiller, but to evaluate the duration and intensity of the effectiveness of acupuncture for the prophylaxis of MWoA in daily clinical practice in China.

## Trial status

This study is currently recruiting patients. This procedure started on 15 October 2012. This trial is anticipated to be completed on 15 September 2014.

## Abbreviations

MWoA: Migraine without aura; NSAIDs: Non-steroidal anti-inflammatory drugs; TCM: Traditional Chinese medicine; RCTs: Randomized controlled trials; IHS: The International Headache Society; CIMS: Clinical Information Management System; SMS: Short message service; CONSORT: The Consolidated Standards of Reporting Trials statement; VAS: Visual analog scale; AE: Adverse event; SAE: Serious adverse event; MSQ: Migraine-Specific Quality-of-Life Questionnaire; SAS: Zung self-Rating anxiety scale; SDS: Zung self-rating depression scale; ITT: Intention-to-treat; PP: Per protocol; ANOVA: Analysis of variance.

## Competing interests

The authors declare that they have no competing interests.

## Authors’ contributions

CJ, ZL, ZH, LY, LFR, YMX, CXR, GB and LYQ participated in the conception and design of the trial. CJ, YMX and ZL drafted the manuscript. All authors approved the final manuscript.
